# Supplementary material for: Spatiotemporal resolution of germinal center Tfh cell differentiation and divergence from central memory CD4+ T cell fate
Source: Nat Commun. 2023 Jun 17;14:3611. doi: 10.1038/s41467-023-39299-3 (PMC10276816; doi:10.1038/s41467-023-39299-3)
Supplement: Supplementary file 1 — Supplementary Information [file 41467_2023_39299_MOESM1_ESM.pdf]

Supplementary Information for

Spatiotemporal resolution of germinal center Tfh cell differentiation and divergence from central memory $CD4^+$ T cell fate

Fangming Zhu[1,8], Ryan J. McMonigle[1,8], Andrew R. Schroeder[1], Xianyou Xia[1], David Figge[2], Braxton D. Greer[3], Edahí González-Avalos[4], Diego O. Sialer[1], Yin-Hu Wang[1], Kelly M. Chandler[1], Adam J. Getzler[5], Emily R. Brown[1], Changchun Xiao[6], Olaf Kutsch[3], Yohsuke Harada[7], Matthew E. Pipkin[5], and Hui Hu[1]*

[1]Department of Microbiology, [2]Department of Pathology, [3]Department of Medicine, School of Medicine, University of Alabama at Birmingham, Birmingham, AL 35294, USA.

[4]Division of Signaling and Gene Expression, La Jolla Institute for Immunology, La Jolla, CA 92037, USA.

[5]Department of Immunology and Microbiology, The Scripps Research Institute, Jupiter, FL 33458, USA.

[6]Department of Immunology and Microbiology, The Scripps Research Institute, La Jolla, CA 92037, USA.

[7]Faculty of Pharmaceutical Sciences, Tokyo University of Science, Chiba 278-8510, Japan.

[8]These authors contributed equally: Fangming Zhu, Ryan J. McMonigle.

*Corresponding author. Email: huihu@uab.edu

Contents

Supplementary Figs. 1 to 8

Supplementary Table 1 to 4

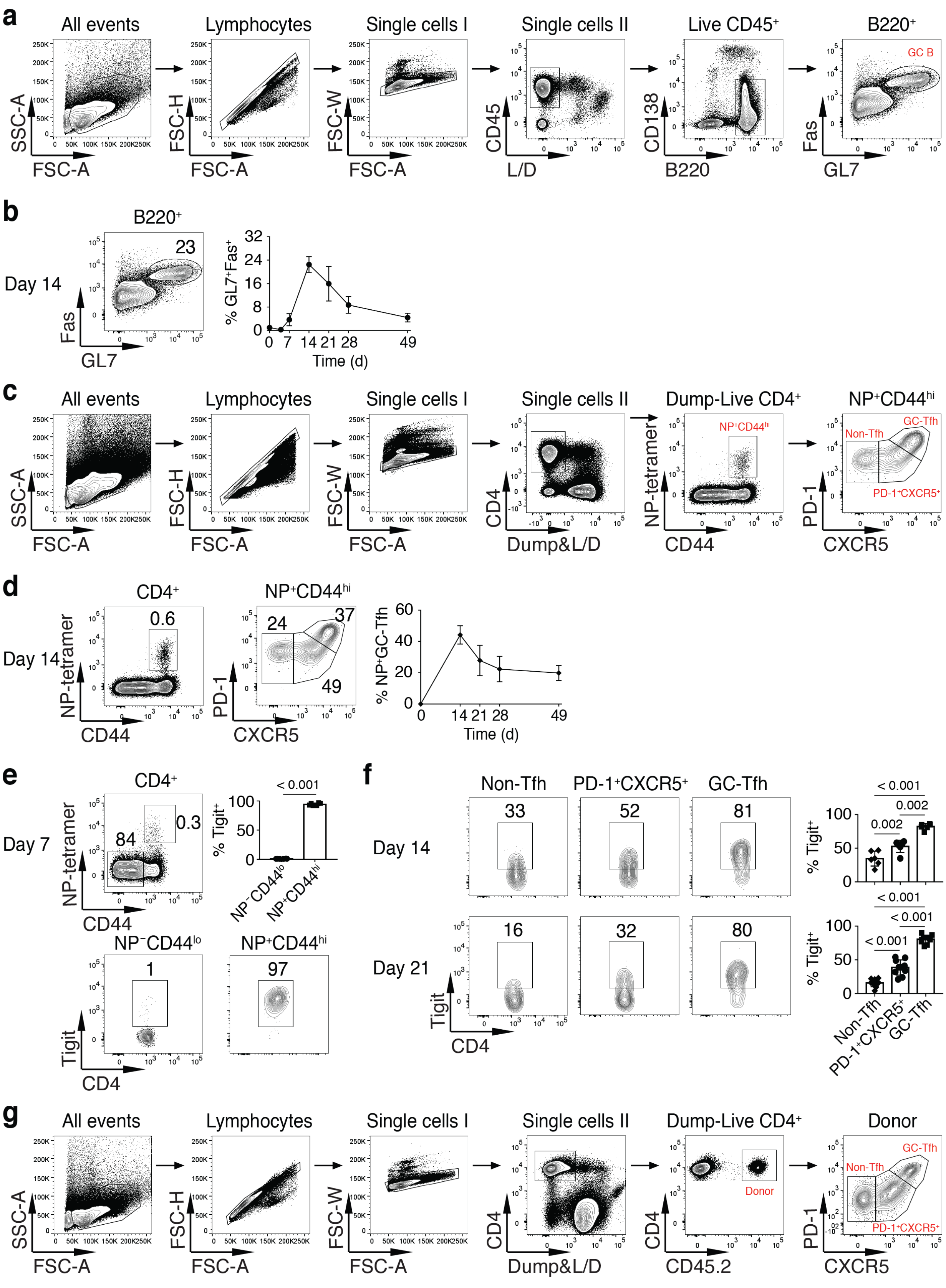
a
All events
Lymphocytes
Single cells I
Single cells II
Live CD45+
B220+
GC B
SSC-A
FSC-A
FSC-H
FSC-W
CD45
L/D
CD138
B220
Fas
GL7
b
B220+
Day 14
23
% GL7+Fas+
Time (d)
c
Dump-Live CD4+
NP+CD44hi
Non-Tfh
GC-Tfh
PD-1+CXCR5+
CD4
Dump&L/D
NP-tetramer
CD44
PD-1
CXCR5
d
CD4+
0.6
24
37
49
% NP+GC-Tfh
e
Day 7
84
0.3
< 0.001
% Tigit+
NP-CD44lo
NP+CD44hi
1
97
Tigit
f
Day 14
Day 21
33
52
81
16
32
80
0.002
< 0.001
g
CD45.2
Donor

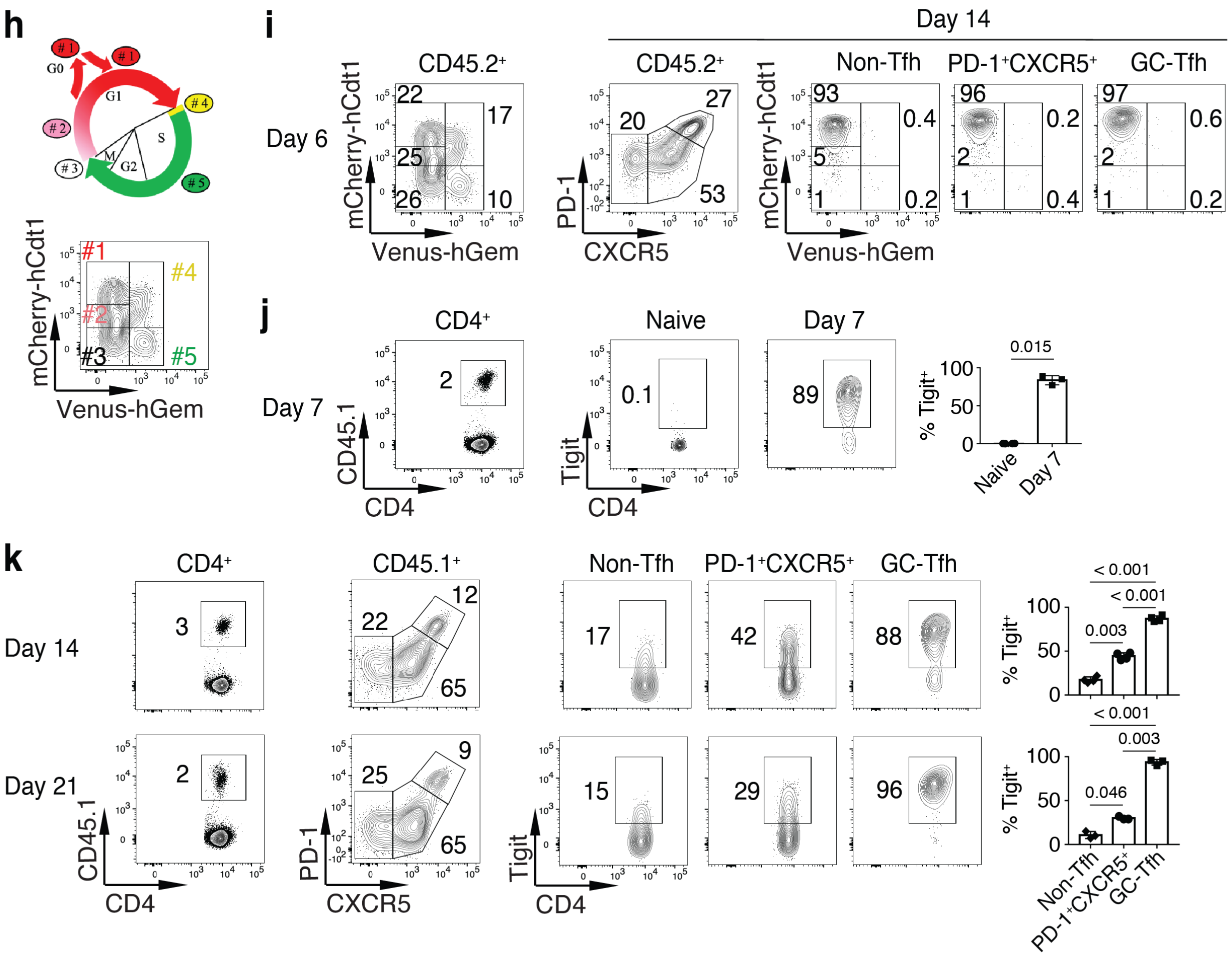

Supplementary Fig. 1 | Analysis of GC response kinetics, GC-Tfh cell proliferation, and Tigit expression. a–f C57BL/6 mice were intranasally infected with PR8. **a** FACS gating strategy for GC B cells. **b** B220$^+$ B cells at days 0, 4, 7, 14, 21, 28, and 49 p.i. in the medLN were analyzed for GL7 and Fas staining. **c** FACS gating strategy for NP$^+$CD44^{hi}CD4$^+$ T cells. **d** NP$^+$CD44^{hi}CD4$^+$ T cells at days 0, 14, 21, 28, and 49 p.i. in the medLN were analyzed for PD-1 and CXCR5 staining (day 0, n = 9; day 4, n = 5; day 7, n = 11; day 14, n = 6; day 21, n = 6; day 28, n = 6; day 49, n = 5). (**e**) NP$^-$CD44^{lo} naïve and NP$^+$CD44^{hi}CD4$^+$ T cells at day 7 p.i. and (**f**) indicated populations of NP$^+$CD44^{hi}CD4$^+$ T cells at days 14 and 21 p.i. in the medLN were analyzed for Tigit staining (day 7, n = 6; day 14, n = 6; day 21, n = 10). **g** Purified OT-II cells were transferred into CD45.1$^+$ SMARTA recipient mice followed by intranasal infection with PR8-OVA. FACS gating strategy for donor OT-II cells. **h** Diagram of Venus-hGem and mCherry-hCdt1 expression throughout the cell cycle in Fucci2 cells (top) and representative flow plot of proliferating OT-II^{Tg} Fucci2 (OT-II-Fucci2) cells (bottom). **i** Purified OT-II cells from

OT-II-Fucci2 mice were transferred into CD45.1$^+$ SMARTA recipient mice followed by intranasal infection with PR8-OVA. Donor OT-II cells at day 6 p.i. (left) and indicated donor cell populations at day 14 p.i. (right) in the medLN were analyzed for Venus-hGem and mCherry-hCdt1 expression (day 6, n = 4; day 14, n = 4). **j, k** Purified CD45.1$^+$ OT-II cells were transferred into CD45.2$^+$ C57BL/6 recipient mice followed by intranasal infection with PR8-OVA. (**j**) Naïve OT-II and donor OT-II at day 7 p.i. and (**k**) indicated donor cell populations at days 14 and 21 p.i. in the medLN were analyzed for Tigit staining (naïve, n = 4; day 7, n = 3; day 14, n = 4; day 21, n = 3). Data in (**b**, **d**–**f**, **i**–**k**) are representative (or pooled) results of at least two independent experiments. Bars represent average ± SD. The *P*-values were determined by a two-tailed unpaired *t*-test (**e**, **j**), or a one-way ANOVA with Tukey's multiple comparisons test (**f**, **k**). Source data are provided as a Source Data file.

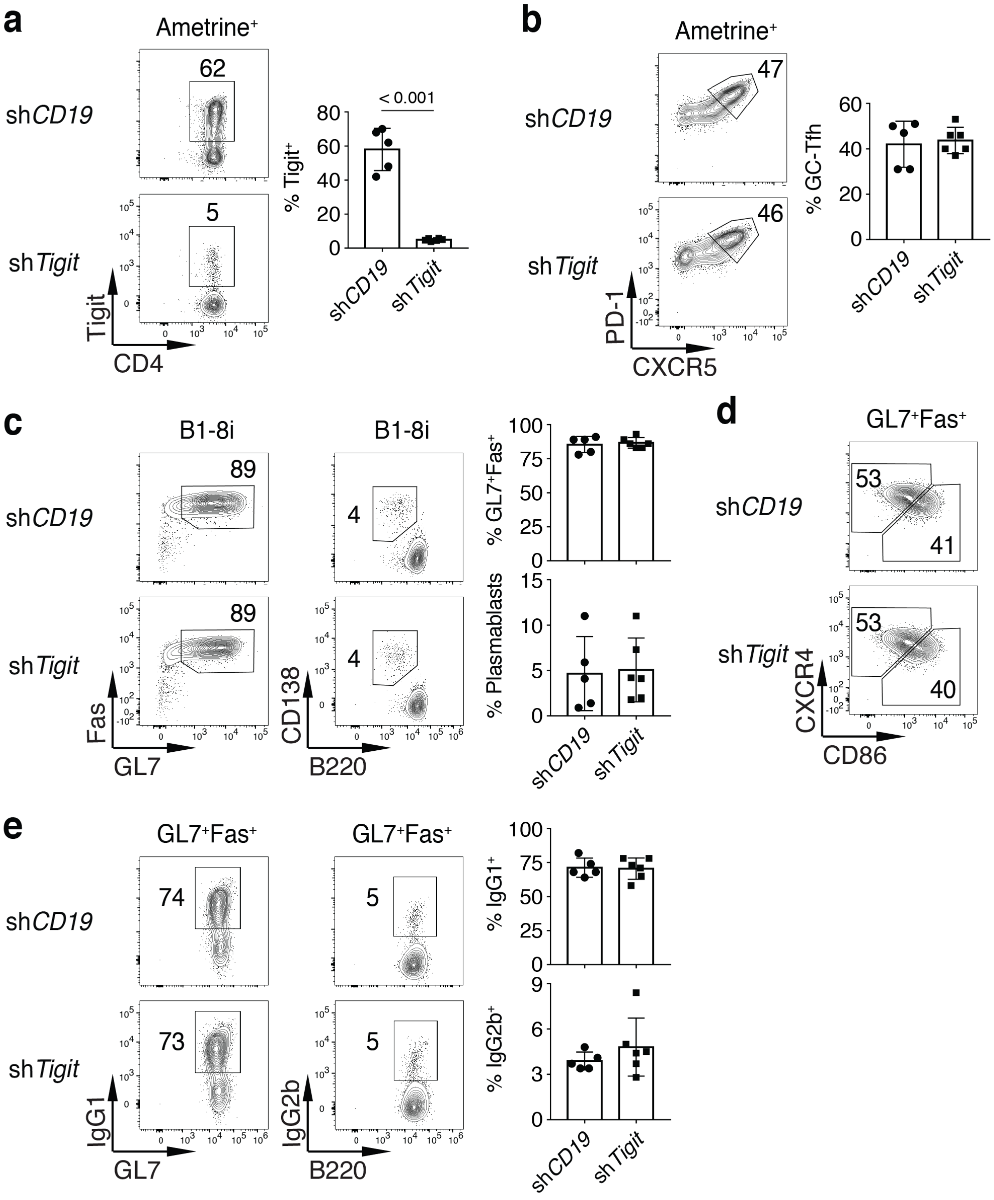

Supplementary Fig. 2 | Tigit signaling is not critical for Tfh cell differentiation or Tfh helper function to B cells. Purified OT-II cells were activated *in vitro* and infected with retrovirus expressing shRNAmir against *CD19* (sh*CD19*) or *Tigit* (sh*Tigit*). Retrovirally infected OT-II cells were co-transferred with B1-8i cells into CD45.1^{+} *Bcl6*$^{f/f}$CD4-Cre^{Tg} recipient mice followed by intranasal immunization with NP-OVA and LPS 1 day after cell transfer. Donor OT-II cells (**a**, **b**) and B1-8i cells (**c**–**e**) at day 6 p.i. in the medLN were analyzed for (**a**) Tigit expression, (**b**) PD-1 and CXCR5 expression gated on Ametrine^{+} donor OT-II cells, (**c**) GC B (GL7^{+}Fas^{+}) cells and plasmablasts (CD138^{+}B220^{int}) of donor B1-8i cells, (**d**) light zone

(CD86^{hi}CXCR4^{lo}) and dark zone (CD86^{lo}CXCR4^{hi}) ratio of donor GC B cells, and (**e**) IgG1 and IgG2b expression of donor GC B cells (sh*CD19*, n = 5; sh*Tigit*, n = 6). Data in (**a**–**e**) are representative results of at least two independent experiments. Bars represent average $\pm$ SD. The *P*-values were determined by a two-tailed unpaired *t*-test (**a**–**e**). Source data are provided as a Source Data file.

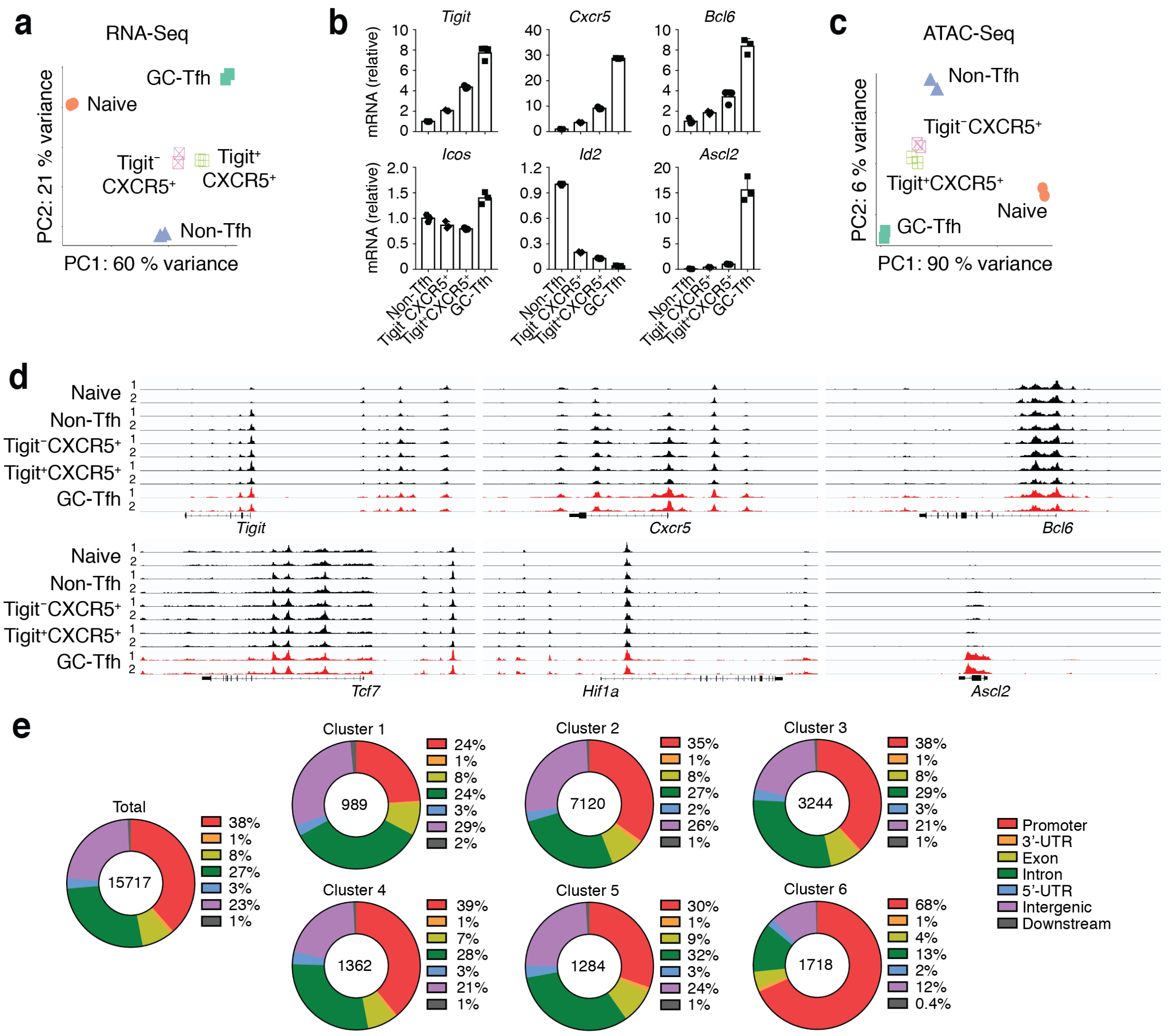

Supplementary Fig. 3 | Analysis of gene expression and chromatin accessibility in Tfh cell differentiation. Purified OT-II cells were transferred into CD45.1^{+} SMARTA recipient mice followed by intranasal infection with PR8-OVA. **a** Principal component analysis (PCA) of RNA-seq samples as in Fig. 3a. **b** Donor OT-II cells at day 21 p.i. in the medLN of recipient mice were analyzed by real time RT-PCR for selected genes (n = 3). (**c**) PCA, (**d**) read density at selected gene loci, and (**e**) doughnut plots of genomic feature analysis for ATAC-seq samples as in Fig. 3d. Data in (**b**) are representative results of two independent experiments. Bars represent average $\pm$ SD. RNA-seq and ATAC-seq samples are independent biological replicates. Source data are provided as a Source Data file.

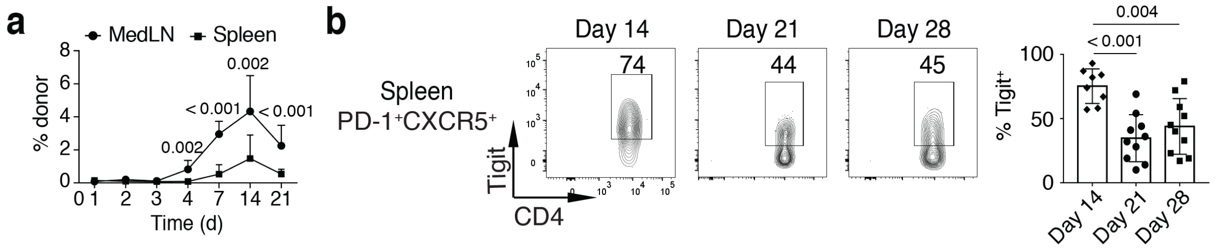

Supplementary Fig. 4 | Kinetics of CD4^{+} T cell expansion and Tigit expression on PD-1^{+}CXCR5^{+}CD4^{+} T cells. Purified CD45.2^{+} OT-II cells were transferred into CD45.1^{+} SMARTA recipient mice followed by intranasal infection with PR8-OVA. **a** The kinetics of donor OT-II cell expansion in the medLN and spleen were analyzed at indicated time points (day 1, n = 2; day 2, n = 2; day 3, n = 2; day 4, n = 8; day 7, n = 7; day 14, n = 10; day 21, n = 14). **b** At days 14, 21, and 28 p.i., the PD-1^{+}CXCR5^{+} donor cells in the spleen were analyzed for Tigit staining (day 14, n = 8; day 21, n = 10; day 28, n = 10). Data in (**a**, **b**) are pooled results of at least two independent experiments. Bars represent average $\pm$ SD. The *P*-values were determined by a two-tailed unpaired *t*-test (**a**), or a one-way ANOVA with Tukey's multiple comparisons test (**b**). Source data are provided as a Source Data file.

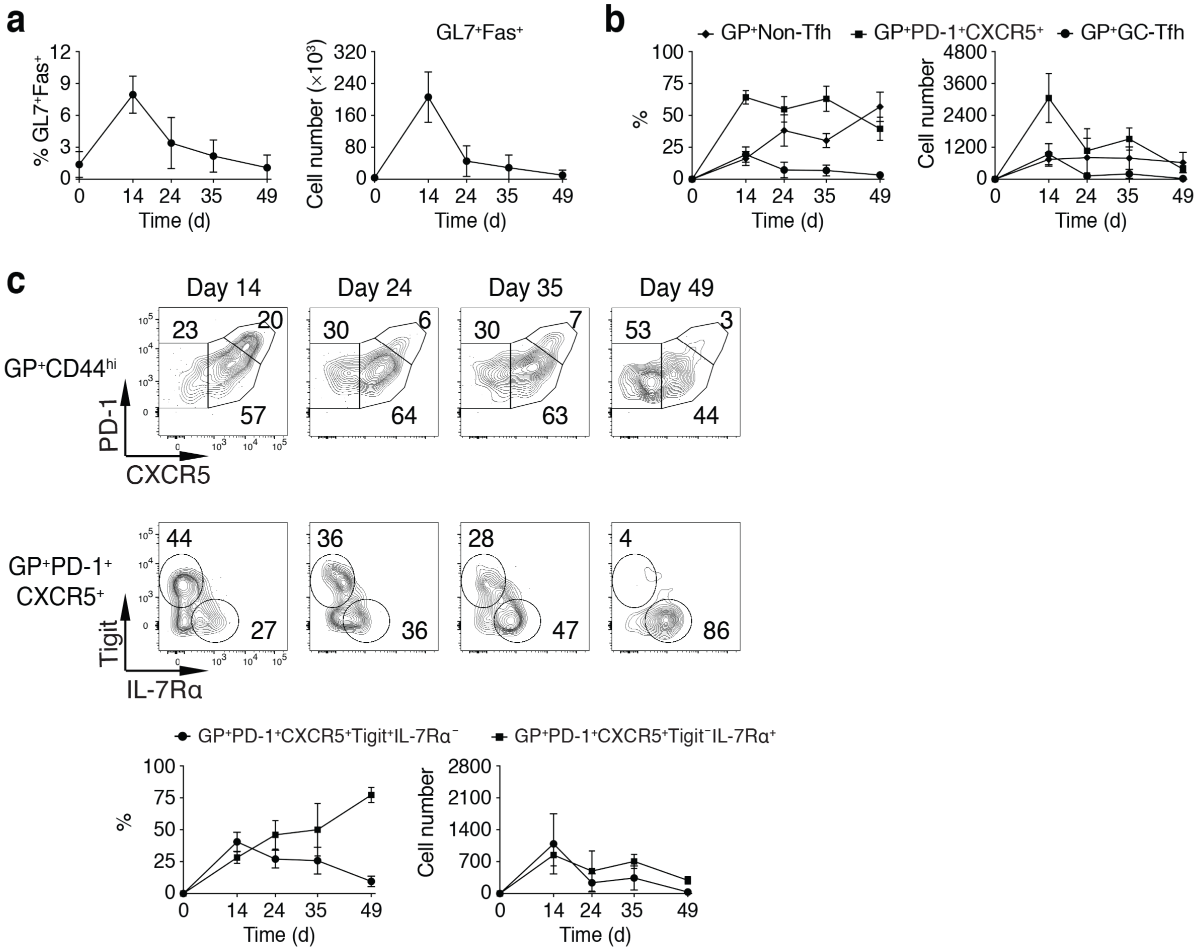

Supplementary Fig. 5 | Kinetics of GC response and Tigit and IL-7Rα expression on PD-1$^+$CXCR5$^+$CD4$^+$ T cells in GP protein immunization model. C57BL/6 mice were intranasally immunized with KLH-GP$_{61-80}$ and LPS. In the medLN at days 0, 14, 24, 35, and 49 p.i., (**a**) B220$^+$ B cells were analyzed for GL7 and FAS staining and GC-B cells (B220$^+$FAS$^+$GL7$^+$) were quantified, (**b**) GP$^+$CD44^{hi}CD4$^+$ T cells were analyzed for PD-1 and CXCR5 staining and GP$^+$ non-Tfh, PD-1$^+$CXCR5$^+$, and GC-Tfh cells were quantified, and (**c**) GP$^+$CD44^{hi}PD-1$^+$CXCR5$^+$CD4$^+$ T cells were analyzed for Tigit and IL-7Rα staining and Tigit$^+$IL-7Rα$^-$ and Tigit$^-$IL-7Rα$^+$ cells were quantified (day 0, n = 8; day 14, n = 6; day 24, n = 5; day 35, n = 4; day 49, n = 4). Data in (**a**–**c**) are representative (or pooled) results of at least two independent experiments. Bars represent average ± SD. Source data are provided as a Source Data file.

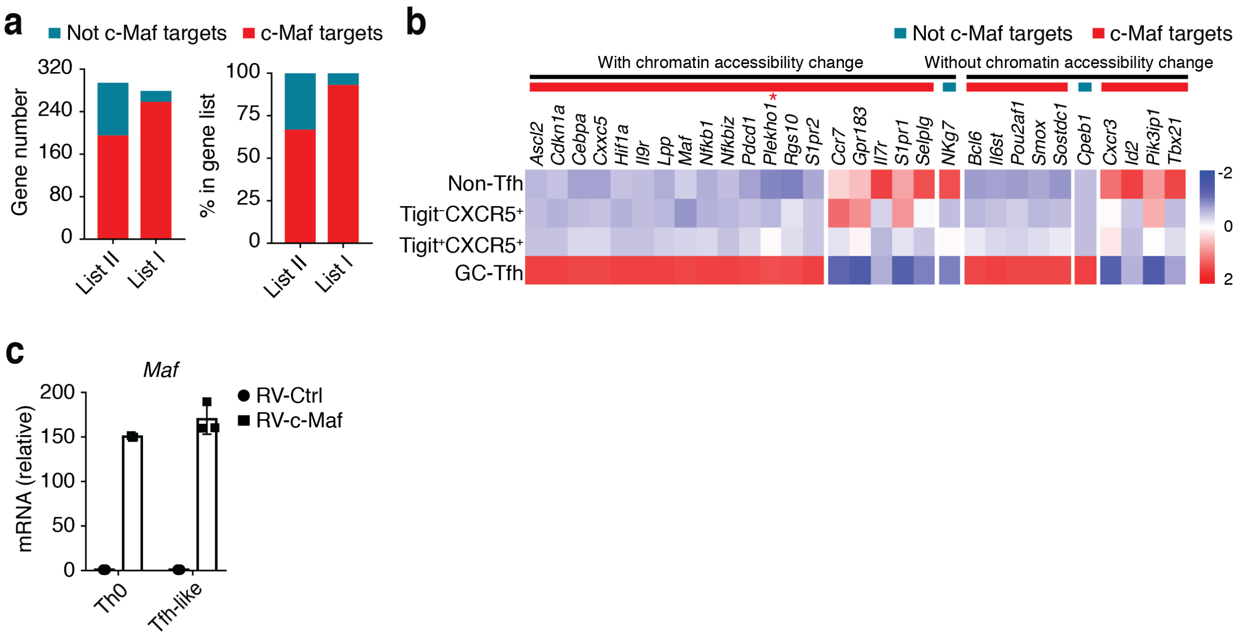

Supplementary Fig. 6 | Cross-examination of potential c-Maf targets with List I and II genes and c-Maf over-expression. a Gene numbers and percentages of potential c-Maf targets in List I and List II genes from Fig. 6a. **b** Expression heatmap of select genes from Supplementary Fig. 6a. **c** Purified $CD4^+$ T cells from C57BL/6 mice were activated *in vitro* and infected with control retrovirus (RV-Ctrl) or retrovirus expressing *Maf* (RV-c-Maf) under Th0 and Tfh-like culture conditions. At day 4, retrovirally infected cells were sorted and analyzed by real time RT-PCR for *Maf* mRNA levels (n = 3). Data in (**c**) are representative results of two independent experiments. Bars represent average ± SD. Source data are provided as a Source Data file.

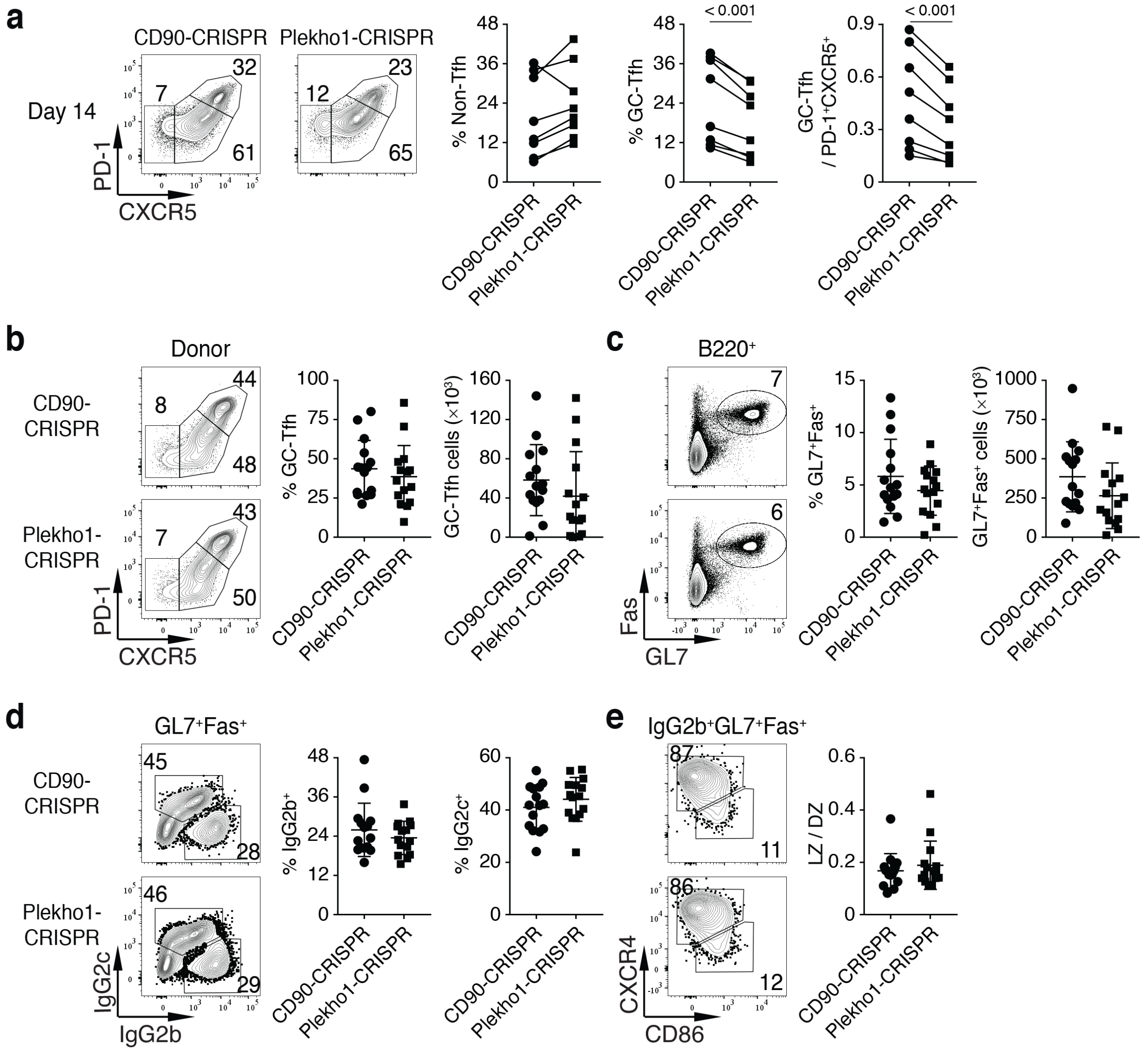

Supplementary Fig. 7 | Function of Plekho1 in Tfh cell differentiation in competitive versus non-competitive environments. a CRISPR/Cas9 was used to delete *Thy1* (CD90-CRISPR) or *Plekho1* (Plekho1-CRISPR) in purified OT-II cells from CD45.1^{+}CD45.2^{+} OT-II^{Tg} or CD45.2^{+} OT-II^{Tg} mice, respectively. CD90-CRISPR and Plekho1-CRISPR OT-II cells were co-transferred into CD45.1^{+} SMARTA recipient mice followed by intranasal infection with PR8-OVA. Donor OT-II cells at day 14 p.i. in the medLN were analyzed for PD-1 and CXCR5 staining (n = 8). **b**–**e** CRISPR/Cas9 was used to delete *Thy1* (CD90-CRISPR) or *Plekho1* (Plekho1-CRISPR) in purified OT-II cells from CD45.2^{+} OT-II^{Tg} mice. CD90-CRISPR or Plekho1-CRISPR OT-II cells were separately transferred into CD45.1^{+} *Bcl6*$^{f/f}$CD4-Cre^{Tg} recipient mice followed by intranasal infection with PR8-OVA. Donor OT-II cells (**b**) and endogenous B cells (**c**–**e**) at day

14 p.i. in the medLN were analyzed for (**b**) PD-1 and CXCR5 staining, (**c**) GC B ($GL7^{+}Fas^{+}$) cells, (**d**) IgG2b and IgG2c expression of GC B cells, and (**e**) light zone ($CD86^{hi}CXCR4^{lo}$) and dark zone ($CD86^{lo}CXCR4^{hi}$) ratios of $IgG2b^{+}$ GC B cells, and GC-Tfh (**b**) and GC B (**c**) cells were quantified (CD90-CRISPR, n = 15; Plekho1-CRISPR, n = 15). Data in (**a**–**e**) are pooled results of two independent experiments. Bars represent average ± SD. The *P*-values were determined by a two-tailed paired *t*-test (**a**), or a two-tailed unpaired *t*-test (**b**–**e**). Source data are provided as a Source Data file.

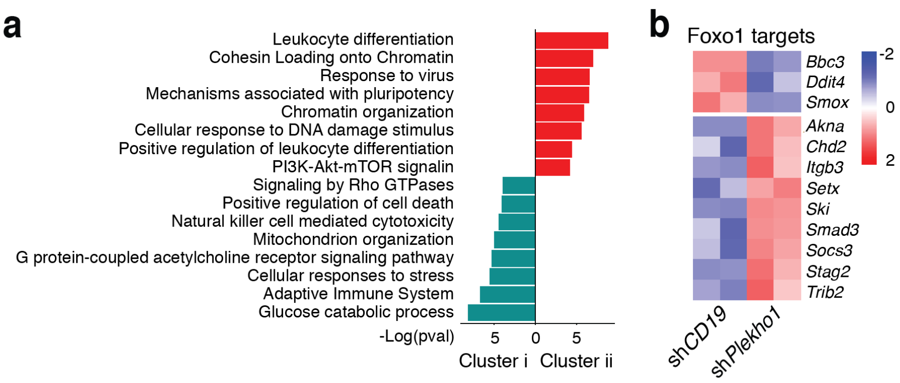

Supplementary Fig. 8 | Metascape pathway analysis and expression heatmap of selected genes. a Metascape pathway enrichment of DEGs in Fig. 7e. **b** Expression heatmap of select genes from Fig. 7e. RNA-seq samples are independent biological replicates. Source data are provided as a Source Data file.

Supplementary Table 1: Virus Strains, Cell Lines, and Organisms/Strains

**Virus Strains**	**Source**	**Identifier**
A/Puerto Rico/8/34	Drs. Frances Lund and Troy Randall	n/a
A/Puerto Rico/8/34-OVA$_{323-339}$	Dr. Paul G. Thomas	n/a
**Cell Lines**		
Plat-E cells	Dr. Matthew Pipkin	n/a
**Organisms/Strains**		
B6 CD45.2	The Jackson Laboratory	C57BL/6J
B6 CD45.1	The Jackson Laboratory	B6.SJL-*Ptprc*^{a} *Pepc*^{b}/BoyJ
B6 CD45.1/CD45.2	Our lab	n/a
OT-II	The Jackson Laboratory	B6.Cg-Tg(TcraTcrb)425Cbn/J
SMARTA	The Jackson Laboratory	B6.Cg-<br>*Ptprc*^{a} *Pepc*^{b} Tg(TcrLCMV)1Aox/PpmJ
B1-8i	The Jackson Laboratory	B6.129P2(C)-*Igh*^{tm2Cgn}/J
Bcl6$^{f/f}$	Dr. Changchun Xiao	n/a
R26p-Fucci2	RIKEN	Tg(Gt(ROSA)26Sor-Fucci2)#Sia
CD4-Cre	The Jackson Laboratory	B6.Cg-Tg(Cd4-cre)1Cwi/BfluJ
Bcl6-tdTomato-Cre-ERT2	Dr. Yohsuke Harada	[1]

Supplementary Table 2: Reagents and Critical Commercial Assays

**Reagents and Critical Commercial Assays**	**Source**	**Identifier**
Polyethylenimine, Linear, MW 25000	Polysciences	Cat#23966-1
Chloroform	Thermo Fisher Scientific	Cat#C298-500
SsoAdvanced Universal SYBR® Green Supermix	Bio-Rad Laboratories	Cat#1725272
Sucrose	AmericanBio	Cat#AB01900
Isopropanol	Thermo Fisher Scientific	Cat#AC327270010
Ethanol	Thermo Fisher Scientific	Cat#BP2818-500
Acetone	Thermo Fisher Scientific	Cat#A18P-4
Paraformaldehyde, EM Grade	Electron Microscopy Sciences	Cat#15714
Triton™ X-100 (Electrophoresis)	Thermo Fisher Scientific	Cat#BP151-500
Sudan Black B	Thermo Fisher Scientific	Cat#J62268.22
Normal Goat Serum	Abcam	Cat#ab7481
ProLong™ Gold Antifade Mountant	Thermo Fisher Scientific	Cat#P36934
rhIL-2	PeproTech	Cat#200-02
IL-6	R&D Systems	Cat#406-ML-005
IL-21	R&D Systems	Cat#594-ML-100
Streptavidin APC	Biolegend	Cat#405207
Streptavidin BV650	Biolegend	Cat#405232
I-A(b) LCMV GP_{66-77} (DIYKGVYQKSV) tetramer-APC	NIH tetramer core facility	n/a
I-A(b) Influenza A $NP_{311-325}$ (QVYSLIRPNENPAHK) tetramer-APC	NIH tetramer core facility	n/a
Lipopolysaccharides from Escherichia coli O55:B5	Sigma	Cat#L2880-25MG
GP_{61}-KLH	Dr. Shane Crotty	[2]
Dynabeads™ Mouse CD4	Thermo Fisher Scientific	Cat#11445D
DETACHaBEAD® Mouse CD4	Thermo Fisher Scientific	Cat#12406D
CD4 (L3T4) MicroBeads, mouse	Miltenyi Biotec	Cat#130-117-043
Streptavidin MicroBeads	Miltenyi Biotec	Cat#130-048-101
LS Columns	Miltenyi Biotec	Cat#130-042-401
TRIzol™ LS Reagent	Life Technologies	Cat#10296010
miRNeasy Micro Kit	QIAGEN	Cat#1071023
SuperScript™ III First-Strand Synthesis System	Thermo Fisher Scientific	Cat#18080051
Nextera DNA Library Prep Kit	Illumina	Cat#15028212
Live/Dead Fixable Blue	Thermo Fisher Scientific	Cat#L23105
Fixable Viability Dye eFluor780	eBioscience	Cat#65-0865-14
DAPI	Biolegend	Cat#422801
Foxp3 / Transcription Factor Staining Buffer Set	eBioscience	Cat#00-5523-00
BD Cytofix/Cytoperm™ Fixation/Permeabilization Solution Kit	BD Biosciences	Cat#554714
TrueCut™ Cas9 Protein v2	Thermo Fisher Scientific	Cat#A36497
P4 Primary Cell 4D-Nucleofector X Kit S	Lonza Bioscience	Cat#V4XP-4032

Supplementary Table 3: Real-time RT-PCR Primers, CRISPR-Cas9 guide RNA, and Recombinant DNA

**Real-time RT-PCR Primers**	**Source**	**Identifier**
Ascl2	n/a	5'-3' Fwd: CCTACTCGTCGGAGGAAAGC<br>5'-3' Rev: AGGTAGGTCCACCAGGAGTC
Bcl6	n/a	5'-3' Fwd: GTGATGACCACAGCCATGTACCTGC<br>5'-3' Rev: CACGACCTCGGTAGGCCATGATG
Cxcr5	n/a	5'-3' Fwd: GACCTTCAACCGTGCCTTTCTC<br>5'-3' Rev: GAACTTGCCCTCAGTCTGTAATCC
Icos	n/a	5'-3' Fwd: CTCACCAAGACCAAGGGAAGC<br>5'-3' Rev: CCACAACGAAAGCTGCACACC
Id2	n/a	5'-3' Fwd: TGGAAATCCTGCAGCACGTCATC<br>5'-3' Rev: GACATAAGCTCAGAAGGGAATTCAGAT
Maf	n/a	5'-3' Fwd: AGCAGTTGGTGACCATGTCG<br>5'-3' Rev: TGGAGATCTCCTGCTTGAGG
Plekho1	n/a	5'-3' Fwd: GGAAAAACCGCTATGTGGTGC<br>5'-3' Rev: CAGGGTGAACTTGCTGTGATT
Tigit	n/a	5'-3' Fwd: GAATGGAACCTGAGGAGTCTCT<br>5'-3' Rev: AGCAATGAAGCTCTCTAGGCT
Rpl32	n/a	5'-3' Fwd: CCCAACATCGGTTATGGGAGCA<br>5'-3' Rev: GATGGCCAGCTGTGCTGC
**CRISPR-Cas9 guide RNA**		
Thy1 sgRNA1	n/a	CCGCCATGAGAATAACACCA
Thy1 sgRNA2	n/a	CCTTGGTGTTATTCTCATGG
Thy1 sgRNA3	n/a	GAGCAGGAGAGCGACGCTGA
Maf sgRNA1	n/a	GATCACGGCGGACACCACGG
Maf sgRNA2	n/a	ATGATGCGGTCGGTCTCCAC
Maf sgRNA3	n/a	CGACCTGCCCACCAGTCCCC
Plekho1 sgRNA1	n/a	CGGACCCAGCCGACCTTCTC
Plekho1 sgRNA2	n/a	CAAGATACGGTTTTTAGCTC
Plekho1 sgRNA3	n/a	TCCAACCCGGGGACACCTCA
**Recombinant DNA**		
pCL-Eco	Addgene	https://www.addgene.org/12371/
LMP-Amt sh*CD19*-RV	Dr. Matthew Pipkin	TGCTGTTGACAGTGAGCGAATGGATAAGTCTGACG<br>ACCTATAGTGAAGCCACAGATGTATAGGTCGTCAG<br>ACTTATCCATGTGCCTACTGCCTCGGA
LMP-Amt sh*Plekho1*-RV	Dr. Matthew Pipkin	TGCTGTTGACAGTGAGCGAAAGGAGGTAAAAGAT<br>GAGAAATAGTGAAGCCACAGATGTATTTCTCATCT<br>TTTACCTCCTTCTGCCTACTGCCTCGGA
LMP-Amt sh*Tigit*-RV	Dr. Matthew Pipkin	TGCTGTTGACAGTGAGCGCAAGAAGTCTATTAGAA<br>TGCATTAGTGAAGCCACAGATGTAATGCATTCTAA<br>TAGACTTCTTTTGCCTACTGCCTCGGA
pMIT-GFP	This paper	Coding sequence for GFP was cloned from pMIG plasmid
pMIG-*Maf*	Dr. Remy Bosselut	[3]

Supplementary Table 4: Software and Algorithms

Software and Algorithms	Source	Identifier
Graphpad Prism v.8.2.1	GraphPad Software	http://www.graphpad.com/ RRID: SCR_002798
FlowJo v.10.7.1	BD	https://www.flowjo.com/solutions/flowjo RRID: SCR_008520
IDEAS v.6.2	Amnis	https://www.luminexcorp.com/?wpdmdl=40160 RRID: SCR_020142
Metascape	[4]	http://metascape.org/gp/index.html#/main/step1 RRID: SCR_016620
Trim Galore! v.0.4.4	Babraham Bioinformatics	http://www.bioinformatics.babraham.ac.uk/projects/trim_galore/ RRID: SCR_011847
STAR v.2.5.4b	[5]	http://code.google.com/p/rna-star/ RRID: SCR_004463
HTSeq-Count v.0.12.3	[6]	http://www-huber.embl.de/users/anders/HTSeq/doc/count.html RRID: SCR_011867
Salmon v.0.14.1	[7]	https://combine-lab.github.io/salmon/ RRID: SCR_017036
R software v.4.0.3	n/a	https://www.r-project.org/
DESeq2 v.1.28.1	[8]	https://bioconductor.org/packages/release/bioc/html/DESeq2.html RRID: SCR_015687
pHeatmap v.1.0.12	n/a	https://www.rdocumentation.org/packages/pheatmap/versions/0.2/topics/pheatmap RRID: SCR_016418
IPA v.60467501	QIAGEN	http://www.ingenuity.com/products/pathways_analysis.html RRID: SCR_008653
GSEA v.4.1.0	[9,10]	http://www.broadinstitute.org/gsea/ RRID: SCR_003199
Bowtie2 v.2.3.3	[11]	http://bowtie-bio.sourceforge.net/bowtie2/index.shtml RRID: SCR_016368
Picard v.2.20.0	Broad Institute	http://broadinstitute.github.io/picard/ RRID: SCR_006525
MACS v.2	[12]	https://github.com/macs3-project/MACS RRID: SCR_013291
IDR v.2.0.3	[13]	https://github.com/nboley/idr RRID: SCR_017237
BedTools v.2.28.0	[14]	https://github.com/arq5x/bedtools2 RRID: SCR_006646
HOMER v.4.11.1	[15]	http://homer.ucsd.edu/ RRID: SCR_010881
IGV v.2.7.2	[16]	http://www.broadinstitute.org/igv/ RRID: SCR_011793

References

1 Takahashi, D. *et al.* Microbiota-derived butyrate limits the autoimmune response by promoting the differentiation of follicular regulatory T cells. *EBioMedicine* **58**, 102913, doi:10.1016/j.ebiom.2020.102913 (2020).

2 Choi, Y. S. *et al.* Bcl6 expressing follicular helper CD4 T cells are fate committed early and have the capacity to form memory. *J Immunol* **190**, 4014-4026, doi:10.4049/jimmunol.1202963 (2013).

3 Vacchio, M. S. *et al.* A Thpok-Directed Transcriptional Circuitry Promotes Bcl6 and Maf Expression to Orchestrate T Follicular Helper Differentiation. *Immunity* **51**, 465-478 e466, doi:10.1016/j.immuni.2019.06.023 (2019).

4 Zhou, Y. *et al.* Metascape provides a biologist-oriented resource for the analysis of systems-level datasets. *Nat Commun* **10**, 1523, doi:10.1038/s41467-019-09234-6 (2019).

5 Dobin, A. *et al.* STAR: ultrafast universal RNA-seq aligner. *Bioinformatics* **29**, 15-21, doi:10.1093/bioinformatics/bts635 (2013).

6 Anders, S., Pyl, P. T. & Huber, W. HTSeq--a Python framework to work with high-throughput sequencing data. *Bioinformatics* **31**, 166-169, doi:10.1093/bioinformatics/btu638 (2015).

7 Patro, R., Duggal, G., Love, M. I., Irizarry, R. A. & Kingsford, C. Salmon provides fast and bias-aware quantification of transcript expression. *Nat Methods* **14**, 417-419, doi:10.1038/nmeth.4197 (2017).

8 Love, M. I., Huber, W. & Anders, S. Moderated estimation of fold change and dispersion for RNA-seq data with DESeq2. *Genome Biol* **15**, 550, doi:10.1186/s13059-014-0550-8 (2014).

9 Mootha, V. K. *et al.* PGC-1alpha-responsive genes involved in oxidative phosphorylation are coordinately downregulated in human diabetes. *Nat Genet* **34**, 267-273, doi:10.1038/ng1180 (2003).

10 Subramanian, A. *et al.* Gene set enrichment analysis: a knowledge-based approach for interpreting genome-wide expression profiles. *Proc Natl Acad Sci U S A* **102**, 15545-15550, doi:10.1073/pnas.0506580102 (2005).

11 Langmead, B. & Salzberg, S. L. Fast gapped-read alignment with Bowtie 2. *Nat Methods* **9**, 357-359, doi:10.1038/nmeth.1923 (2012).

12 Zhang, Y. *et al.* Model-based analysis of ChIP-Seq (MACS). *Genome Biol* **9**, R137, doi:10.1186/gb-2008-9-9-r137 (2008).

13 Li, Q. H., Brown, J. B., Huang, H. Y. & Bickel, P. J. Measuring Reproducibility of High-Throughput Experiments. *Ann Appl Stat* **5**, 1752-1779, doi:10.1214/11-Aoas466 (2011).

14 Quinlan, A. R. & Hall, I. M. BEDTools: a flexible suite of utilities for comparing genomic features. *Bioinformatics* **26**, 841-842, doi:10.1093/bioinformatics/btq033 (2010).

15 Heinz, S. *et al.* Simple combinations of lineage-determining transcription factors prime cis-regulatory elements required for macrophage and B cell identities. *Mol Cell* **38**, 576-589, doi:10.1016/j.molcel.2010.05.004 (2010).

16 Robinson, J. T. *et al.* Integrative genomics viewer. *Nat Biotechnol* **29**, 24-26, doi:10.1038/nbt.1754 (2011).
