# Supplementary material for: Spatiotemporal resolution of germinal center Tfh cell differentiation and divergence from central memory CD4+ T cell fate
Source: Nat Commun. 2023 Jun 17;14:3611. doi: 10.1038/s41467-023-39299-3 (PMC10276816; doi:10.1038/s41467-023-39299-3)
Supplement: Supplementary file 3 — Reporting Summary [file 41467_2023_39299_MOESM3_ESM.pdf]

nature portfolio

Corresponding author(s): Hui Hu

Last updated by author(s): May 20, 2023

Reporting Summary

Nature Portfolio wishes to improve the reproducibility of the work that we publish. This form provides structure for consistency and transparency in reporting. For further information on Nature Portfolio policies, see our Editorial Policies and the Editorial Policy Checklist.

Statistics

For all statistical analyses, confirm that the following items are present in the figure legend, table legend, main text, or Methods section.

n/a	Confirmed	
☐	☒	The exact sample size (*n*) for each experimental group/condition, given as a discrete number and unit of measurement
☐	☒	A statement on whether measurements were taken from distinct samples or whether the same sample was measured repeatedly
☐	☒	The statistical test(s) used AND whether they are one- or two-sided<br>*Only common tests should be described solely by name; describe more complex techniques in the Methods section.*
☒	☐	A description of all covariates tested
☒	☐	A description of any assumptions or corrections, such as tests of normality and adjustment for multiple comparisons
☐	☒	A full description of the statistical parameters including central tendency (e.g. means) or other basic estimates (e.g. regression coefficient) AND variation (e.g. standard deviation) or associated estimates of uncertainty (e.g. confidence intervals)
☐	☒	For null hypothesis testing, the test statistic (e.g. *F*, *t*, *r*) with confidence intervals, effect sizes, degrees of freedom and *P* value noted<br>*Give P values as exact values whenever suitable.*
☒	☐	For Bayesian analysis, information on the choice of priors and Markov chain Monte Carlo settings
☒	☐	For hierarchical and complex designs, identification of the appropriate level for tests and full reporting of outcomes
☒	☐	Estimates of effect sizes (e.g. Cohen's *d*, Pearson's *r*), indicating how they were calculated

Our web collection on statistics for biologists contains articles on many of the points above.

Software and code

Policy information about availability of computer code

Data collection	BD LSR II, BD FACSymphony A3, BD FACSAria II, Amnis ImageStreamX Mark II, Nikon A1R confocal microscope, Illumina NovaSeq 6000
Data analysis	RNA-seq: Data analysis: the Cheaha Supercomputer at the University of Alabama at Birmingham, Trim Galore! (v.0.4.4), STAR (v.2.5.4b), HTSeq-Count (v.0.12.3), Salmon (v.0.14.1), R software (v.4.0.3), DESeq2 (v.1.28.1), pHeatmap (v.1.0.12), IPA (v.60467501; Ingenuity Systems; Qiagen, Redwood City, California), GSEA (v.4.1.0), Metascape (https://metascape.org/gp/index.html#/main/step1), Enrichr (https://maayanlab.cloud/Enrichr/).<br><br>ATAC-seq: the Cheaha Supercomputer at the University of Alabama at Birmingham, Trim Galore! (v.0.4.4), Bowtie2 (v.2.3.3), Picard (v.2.20.0), MACS v2 (FDR q-value 0.01), IDR (v.2.0.3), BedTools (v.2.28.0), R software (v.4.0.3), DESeq2 (v.1.28.1), pHeatmap (v.1.0.12), HOMER (v.4.11.1, default parameters), IGV (v.2.7.2).<br><br>Flow cytometry results were analyzed using FlowJo software (v.10.7.1).<br><br>Imaging flow cytometry results were analyzed using IDEAS software (v.6.2)<br><br>Independent and paired two-tailed Student's t-tests, independent and repeated measure one-way ANOVAs, and repeated measure two-way ANOVAs were performed using GraphPad Prism software (v.8.2.1).<br><br>No custom code was developed in the study.

For manuscripts utilizing custom algorithms or software that are central to the research but not yet described in published literature, software must be made available to editors and reviewers. We strongly encourage code deposition in a community repository (e.g. GitHub). See the Nature Portfolio guidelines for submitting code & software for further information.

Data

Policy information about availability of data

All manuscripts must include a data availability statement. This statement should provide the following information, where applicable:

- Accession codes, unique identifiers, or web links for publicly available datasets
- A description of any restrictions on data availability
- For clinical datasets or third party data, please ensure that the statement adheres to our policy

RNA-seq and ATAC-seq data generated in this study have been deposited at GEO (SuperSeries accession number: GSE174104, https://www.ncbi.nlm.nih.gov/geo/query/acc.cgi?&acc=GSE174104). The authors declare that data supporting the findings of this study are available within the paper and its supplementary information files. Source data are provided with this paper.

Human research participants

Policy information about studies involving human research participants and Sex and Gender in Research.

Reporting on sex and gender	N/A
Population characteristics	N/A
Recruitment	N/A
Ethics oversight	N/A

Note that full information on the approval of the study protocol must also be provided in the manuscript.

Field-specific reporting

Please select the one below that is the best fit for your research. If you are not sure, read the appropriate sections before making your selection.

☒ Life sciences ☐ Behavioural & social sciences ☐ Ecological, evolutionary & environmental sciences

For a reference copy of the document with all sections, see nature.com/documents/nr-reporting-summary-flat.pdf

Life sciences study design

All studies must disclose on these points even when the disclosure is negative.

Sample size	Samples sizes were not predetermined. Samples sizes were chosen based on a previous study from our lab. Generally, we used at least 3 biological samples to achieves 95% power for detecting statistical difference.
Data exclusions	No data points were excluded.
Replication	All experimental findings were reliably reproduced. We performed at least two independent biological replicates of each assay and all results were reproducible.
Randomization	Experiments were conducted using age and sex matched male and female mice at 6-12 weeks of age.
Blinding	Investigators were not blinded to group allocation during data collection or analysis to avoid any mislabeling of different groups.

Reporting for specific materials, systems and methods

We require information from authors about some types of materials, experimental systems and methods used in many studies. Here, indicate whether each material, system or method listed is relevant to your study. If you are not sure if a list item applies to your research, read the appropriate section before selecting a response.

Materials & experimental systems

n/a	Involved in the study
☐	☒ Antibodies
☐	☒ Eukaryotic cell lines
☒	☐ Palaeontology and archaeology
☐	☒ Animals and other organisms
☒	☐ Clinical data
☒	☐ Dual use research of concern

Methods

n/a	Involved in the study
☒	☐ ChIP-seq
☐	☒ Flow cytometry
☒	☐ MRI-based neuroimaging

Antibodies

Antibodies used

anti-mouse CD4 FITC (Clone RM4-5) Biolegend 1:400 Cat#116004; RRID: AB_313689
anti-mouse CD4 PerCP-Cy5.5 (Clone GK1.5) Biolegend 1:400 Cat#100434; RRID: AB_893324
anti-mouse CD4 BV421 (Clone GK1.5) Biolegend 1:800 Cat#100443; RRID: AB_2562557
anti-mouse CD4 BV605 (Clone RM4-5) Biolegend 1:400 Cat#100548; RRID: AB_2563054
anti-mouse CD4 BV650 (Clone RM4-5) Biolegend 1:400 Cat#100546; RRID: AB_2562098
anti-mouse CD4 APC (Clone GK1.5) Biolegend 1:400 Cat#100412; RRID: AB_312697
anti-mouse CD4 APC-Cy7 (Clone GK1.5) Biolegend 1:400 Cat#100414; RRID: AB_312699
anti-mouse CD8a PerCP-Cy5.5 (Clone 53-6.7) Biolegend 1:400 Cat#100734; RRID: AB_2075238
anti-mouse CD8a APC-Cy7 (Clone 53-6.7) Biolegend 1:400 Cat#100714; RRID: AB_312753
anti-mouse/human CD11b PerCP-Cy5.5 (Clone M1/70) Biolegend 1:800 Cat#101228; RRID: AB_893232
anti-mouse/human CD11b APC-Cy7 (Clone M1/70) Biolegend 1:800 Cat#101226; RRID: AB_830642
anti-mouse CD11c PerCP-Cy5.5 (Clone N418) Biolegend 1:800 Cat#117328; RRID: AB_2129641
anti-mouse CD11c APC-Cy7 (Clone N418) Biolegend 1:800 Cat#117324; RRID: AB_830649
anti-mouse CD16/CD32 (Clone 93) Biolegend 3 μg/ml Cat#101302; RRID: AB_312801
anti-mouse CD19 PerCP-Cy5.5 (Clone 6D5) Biolegend 1:400 Cat#115534; RRID: AB_2072925
anti-mouse CD19 BV510 (Clone 6D5) Biolegend 1:400 Cat#115546; RRID: AB_2562137
anti-mouse CD19 APC-Cy7 (Clone 6D5) Biolegend 1:400 Cat#115530; RRID: AB_830707
anti-mouse/human CD44 BV785 (Clone IM7) Biolegend 1:400 Cat#103059; RRID: AB_2571953
anti-mouse/human CD44 APC (Clone IM7) Biolegend 1:400 Cat#103012; RRID: AB_312963
anti-mouse/human CD44 AF700 (Clone IM7) Biolegend 1:200 Cat#103026; RRID: AB_493713
anti-mouse CD45.1 FITC (Clone A20) Biolegend 1:400 Cat#110706; RRID: AB_313495
anti-mouse CD45.1 PerCP-Cy5.5 (Clone A20) Biolegend 1:200 Cat#110728; RRID: AB_893346
anti-mouse CD45.1 APC (Clone A20) Biolegend 1:400 Cat#110714; RRID: AB_313503
anti-mouse CD45.1 AF700 (Clone A20) Biolegend 1:200 Cat#110724; RRID: AB_493733
anti-mouse CD45.2 AF488 (Clone 104) Biolegend 1:400 Cat#109816; RRID: AB_492868
anti-mouse CD45.2 FITC (Clone 104) Biolegend 1:400 Cat#109806; RRID: AB_313443
anti-mouse CD45.2 PerCP-Cy5.5 (Clone 104) Biolegend 1:200 Cat#109828; RRID: AB_893350
anti-mouse CD45.2 PE (Clone 104) eBioscience 1:400 Cat#12-0454-82; RRID: AB_465678
anti-mouse CD45.2 AF700 (Clone 104) Biolegend 1:200 Cat#109822; RRID: AB_493731
anti-mouse CD45.2 APC (Clone 104) Biolegend 1:400 Cat#109814; RRID: AB_389211
anti-mouse/human CD45R/B220 PerCP-Cy5.5 (Clone RA3-6B2) Biolegend 1:400 Cat#103212; RRID: AB_312997
anti-mouse/human CD45R/B220 BV510 (Clone RA3-6B2) Biolegend 1:400 Cat#103248; RRID: AB_2650679
anti-mouse CD62L PerCP-Cy5.5 (Clone MEL-14) Biolegend 1:200 Cat#104432; RRID: AB_2285839
anti-mouse CD62L APC (Clone MEL-14) Biolegend 1:200 Cat#104412; RRID: AB_313099
anti-mouse CD90.2 (Thy-1.2) PerCP-Cy5.5 (Clone 30-H12) Biolegend 1:800 Cat# 105338; RRID: AB_2571945
anti-mouse CD95 (Fas) PE-Cy7 (Clone Jo2) BD Biosciences 1:200 Cat#557653; RRID: AB_396768
anti-mouse CD127 (IL-7Ralpha) FITC (Clone A7R34) Biolegend 1:100 Cat#135008; RRID: AB_1937232
anti-mouse CD127 (IL-7Ralpha) AF647 (Clone A7R34) Biolegend 1:100 Cat#135020; RRID: AB_1937209
anti-mouse CD162 (Psgl-1) BV510 (Clone 2PH1) BD Biosciences 1:400 Cat#563448; RRID: AB_2738211
anti-mouse CD185 (CXCR5) PE (Clone SPRCL5) Thermo Fisher Scientific 1:100 Cat# 13-7185-82; RRID: AB_2572800
anti-mouse CD185 (CXCR5) BV421 (Clone L138D7) Biolegend 1:100 Cat#145512; RRID: AB_2562128
anti-mouse CD186 (CXCR6) PE (Clone SA051D1) Biolegend 1:200 Cat#151104; RRID: AB_2566546
anti-mouse CD197 (CCR7) AF488 (Clone 4B12) Biolegend 1:100 Cat#120110; RRID: AB_492841
anti-Mouse CD197 (CCR7) PE (Clone 4B12) BD Biosciences 1:100 Cat#560682; RRID: AB_1727442
anti-mouse CD279 (PD-1) PE-Cy7 (Clone 29F.1A12) Biolegend 1:400 Cat#135216; RRID: AB_10689635
anti-mouse/human Bcl6 PE (Clone K112-91) BD Biosciences 1:50 Cat#561522; RRID: AB_10717126
anti-mouse/human c-Maf PE (Clone T54-853) BD Biosciences 1:200 Cat#565795; RRID: AB_2739359
anti-mouse/human GL7 AF488 (Clone GL7) Biolegend 1:400 Cat#144612; RRID: AB_2563285
anti-mouse/human GL7 PerCP-Cy5.5 (Clone GL7) Biolegend 1:200 Cat#144610; RRID: AB_2562979
anti-mouse/human GL7 PB (Clone GL7) Biolegend 1:200 Cat#144614; RRID: AB_2563292
anti-mouse IgD AF647 (Clone 11-26c.2a) Biolegend 1:300 Cat#405708; RRID: AB_893528
anti-rabbit IgG (min x-reactivity) DyLight 488 (Clone Poly4064) Biolegend 1:400 Cat#406404; RRID: AB_1575130
anti-mouse Ly-6C PerCP-Cy5.5 (Clone HK1.4) Biolegend 1:400 Cat#128012; RRID: AB_1659241
anti-mouse TCR-beta chain APC-Cy7 (Clone H57-597) BD Biosciences 1:400 Cat#560656; RRID: AB_1727574
anti-mouse Tigit (Vstm3) PE (Clone 1G9) Biolegend 1:200 Cat#142104; RRID: AB_10933258
Biotin anti-mouse Tigit (Vstm3) (Clone 1G9) Biolegend 1:200 Cat#142113; RRID: AB_2687312
Biotin anti-mouse CD45.2 (Clone 104) Biolegend 1:400 Cat# 109804; RRID: AB_313441
anti-Foxo1 (Clone C29H4) Cell Signaling Technology 1:100 Cat#2880; RRID: AB_2106495
anti-mouse Tigit (Polyclonal) R&D 1:200 Cat#AF7267; RRID: AB_10973473

anti-ICOS (Clone C398.4A) Biolegend 5 μg/ml Cat# 313502; RRID: AB_416326
anti-mouse/human CD3 (Clone 145-2C11) eBioscience 0.5 μg/ml Cat# 16-0031-82; RRID: AB_468847
anti-mouse CD28 (Clone 37.51) eBioscience 1 μg/ml Cat#14-0281-82; RRID: AB_467190
anti-Hamster IgG (Polyclonal) Thermo Fisher Scientific 20 μg/ml Cat#ICN56984; RRID: AB_2334783
anti-IL-4 BD Biosciences 10 μg/ml Cat#559062; RRID: AB_397187
anti-IFN-γ Bio X Cell 10 μg/ml Cat#BE0055; RRID: AB_1107694
anti-TGFβ Bio X Cell 10 μg/ml Cat#BE0057; RRID: AB_1107757
Streptavidin APC Biolegend 1:500 Cat#405207
Streptavidin BV650 Biolegend 1:500 Cat#405232
I-A(b) LCMV GP66-77 (DIYKGVYQKSV) tetramer-APC NIH tetramer core facility 1:100 N/A
I-A(b) Influenza A NP311-325 (QVYSLIRPNENPAHK) tetramer-APC NIH tetramer core facility 1:100 N/A

Validation

anti-mouse CD4 FITC (Clone RM4-5) Biolegend 1:400 https://www.biolegend.com/en-us/products/fitc-anti-mouse-cd4-antibody-475
anti-mouse CD4 PerCP-Cy5.5 (Clone GK1.5) Biolegend 1:400 https://www.biolegend.com/en-us/products/percp-cyanine5-5-anti-mouse-cd4-antibody-4220
anti-mouse CD4 BV421 (Clone GK1.5) Biolegend 1:800 https://www.biolegend.com/en-us/products/brilliant-violet-421-anti-mouse-cd4-antibody-7142
anti-mouse CD4 BV605 (Clone RM4-5) Biolegend 1:400 https://www.biolegend.com/en-us/products/brilliant-violet-605-anti-mouse-cd4-antibody-7627
anti-mouse CD4 BV650 (Clone RM4-5) Biolegend 1:400 https://www.biolegend.com/en-us/products/brilliant-violet-650-anti-mouse-cd4-antibody-7634
anti-mouse CD4 APC (Clone GK1.5) Biolegend 1:400 https://www.biolegend.com/en-us/products/apc-anti-mouse-cd4-antibody-245
anti-mouse CD4 APC-Cy7 (Clone GK1.5) Biolegend 1:400 https://www.biolegend.com/en-us/products/apc-cyanine7-anti-mouse-cd4-antibody-1964
anti-mouse CD8a PerCP-Cy5.5 (Clone 53-6.7) Biolegend 1:400 https://www.biolegend.com/en-us/products/percp-cyanine5-5-anti-mouse-cd8a-antibody-4255
anti-mouse CD8a APC-Cy7 (Clone 53-6.7) Biolegend 1:400 https://www.biolegend.com/en-us/products/apc-cyanine7-anti-mouse-cd8a-antibody-2269
anti-mouse/human CD11b PerCP-Cy5.5 (Clone M1/70) Biolegend 1:800 https://www.biolegend.com/en-us/products/percp-cyanine5-5-anti-mouse-human-cd11b-antibody-4257
anti-mouse/human CD11b APC-Cy7 (Clone M1/70) Biolegend 1:800 https://www.biolegend.com/en-us/products/apc-cyanine7-anti-mouse-human-cd11b-antibody-3930
anti-mouse CD11c PerCP-Cy5.5 (Clone N418) Biolegend 1:800 https://www.biolegend.com/en-us/products/percp-cyanine5-5-anti-mouse-cd11c-antibody-4258
anti-mouse CD11c APC-Cy7 (Clone N418) Biolegend 1:800 https://www.biolegend.com/en-us/products/apc-cyanine7-anti-mouse-cd11c-antibody-3931
anti-mouse CD16/CD32 (Clone 93) Biolegend 3 μg/ml https://www.biolegend.com/en-us/products/purified-anti-mouse-cd16-32-antibody-190?GroupID=BLG9237
anti-mouse CD19 PerCP-Cy5.5 (Clone 6D5) Biolegend 1:400 https://www.biolegend.com/en-us/products/percp-cyanine5-5-anti-mouse-cd19-antibody-4261
anti-mouse CD19 BV510 (Clone 6D5) Biolegend 1:400 https://www.biolegend.com/en-us/products/brilliant-violet-510-anti-mouse-cd19-antibody-8563
anti-mouse CD19 APC-Cy7 (Clone 6D5) Biolegend 1:400 https://www.biolegend.com/en-us/products/apc-cyanine7-anti-mouse-cd19-antibody-3903
anti-mouse/human CD44 BV785 (Clone IM7) Biolegend 1:400 https://www.biolegend.com/en-us/products/brilliant-violet-785-anti-mouse-human-cd44-antibody-7959
anti-mouse/human CD44 APC (Clone IM7) Biolegend 1:400 https://www.biolegend.com/en-us/products/apc-anti-mouse-human-cd44-antibody-312
anti-mouse/human CD44 AF700 (Clone IM7) Biolegend 1:200 https://www.biolegend.com/en-us/products/alexa-fluor-700-anti-mouse-human-cd44-antibody-3406
anti-mouse CD45.1 FITC (Clone A20) Biolegend 1:400 https://www.biolegend.com/en-us/products/fitc-anti-mouse-cd45-1-antibody-198
anti-mouse CD45.1 PerCP-Cy5.5 (Clone A20) Biolegend 1:200 https://www.biolegend.com/en-us/products/percp-cyanine5-5-anti-mouse-cd45-1-antibody-4269
anti-mouse CD45.1 APC (Clone A20) Biolegend 1:400 https://www.biolegend.com/en-us/products/apc-anti-mouse-cd45-1-antibody-2319
anti-mouse CD45.1 AF700 (Clone A20) Biolegend 1:200 https://www.biolegend.com/en-us/products/alexa-fluor-700-anti-mouse-cd45-1-antibody-3392
anti-mouse CD45.2 AF488 (Clone 104) Biolegend 1:400 https://www.biolegend.com/en-us/products/alexa-fluor-488-anti-mouse-cd45-2-antibody-3106
anti-mouse CD45.2 FITC (Clone 104) Biolegend 1:400 https://www.biolegend.com/en-us/products/fitc-anti-mouse-cd45-2-antibody-6
anti-mouse CD45.2 PerCP-Cy5.5 (Clone 104) Biolegend 1:200 https://www.biolegend.com/en-us/products/percp-cyanine5-5-anti-mouse-cd452-antibody-4271
anti-mouse CD45.2 PE (Clone 104) eBioscience 1:400 https://www.thermofisher.com/antibody/product/CD45-2-Antibody-clone-104-Monoclonal/12-0454-82
anti-mouse CD45.2 AF700 (Clone 104) Biolegend 1:200 https://www.biolegend.com/en-us/products/alexa-fluor-700-anti-mouse-cd45-2-antibody-3393
anti-mouse CD45.2 APC (Clone 104) Biolegend 1:400 https://www.biolegend.com/en-us/products/apc-anti-mouse-cd45-2-antibody-2759
anti-mouse/human CD45R/B220 PerCP-Cy5.5 (Clone RA3-6B2) Biolegend 1:400 https://www.biolegend.com/en-us/products/percp-cyanine5-5-anti-mouse-human-cd45r-b220-antibody-4267?GroupID=BLG6847
anti-mouse/human CD45R/B220 BV510 (Clone RA3-6B2) Biolegend 1:400 https://www.biolegend.com/en-us/products/brilliant-violet-510-anti-mouse-human-cd45r-b220-antibody-7996
anti-mouse CD62L PerCP-Cy5.5 (Clone MEL-14) Biolegend 1:200 https://www.biolegend.com/en-us/clone-search/percp-cyanine5-5-anti-mouse-cd62l-antibody-4272?GroupID=BLG10534
anti-mouse CD62L APC (Clone MEL-14) Biolegend 1:200 https://www.biolegend.com/en-us/products/apc-anti-mouse-cd62l-

antibody-381
anti-mouse CD90.2 (Thy-1.2) PerCP-Cy5.5 (Clone 30-H12) Biolegend 1:800 https://www.biolegend.com/en-us/products/percp-cyanine5-5-anti-mouse-cd902-thy-12-antibody-8993
anti-mouse CD95 (Fas) PE-Cy7 (Clone Jo2) BD Biosciences 1:200 https://www.bdbiosciences.com/en-us/products/reagents/flow-cytometry-reagents/research-reagents/single-color-antibodies-ruo/pe-cy-7-hamster-anti-mouse-cd95.557653
anti-mouse CD127 (IL-7Ralpha) FITC (Clone A7R34) Biolegend 1:100 https://www.biolegend.com/en-us/products/fitc-anti-mouse-cd127-il-7ralpha-antibody-6189
anti-mouse CD127 (IL-7Ralpha) AF647 (Clone A7R34) Biolegend 1:100 https://www.biolegend.com/en-us/products/alexa-fluor-647-anti-mouse-cd127-il-7ralpha-antibody-6195
anti-mouse CD162 (Psgl-1) BV510 (Clone 2PH1) BD Biosciences 1:400 https://www.biolegend.com/en-us/products/brilliant-violet-510-anti-mouse-cd62l-antibody-8162?GroupID=BLG10534
anti-mouse CD185 (CXCR5) PE (Clone SPRCL5) Thermo Fisher Scientific 1:100 https://www.thermofisher.com/antibody/product/CD185-CXCR5-Antibody-clone-SPRCL5-Monoclonal/12-7185-82
anti-mouse CD185 (CXCR5) BV421 (Clone L138D7) Biolegend 1:100 https://www.biolegend.com/en-us/products/brilliant-violet-421-anti-mouse-cd185-cxcr5-antibody-8553
anti-mouse CD186 (CXCR6) PE (Clone SA051D1) Biolegend 1:200 https://www.biolegend.com/en-us/products/pe-anti-mouse-cd186-cxcr6-antibody-12545
anti-mouse CD197 (CCR7) AF488 (Clone 4B12) Biolegend 1:100 https://www.biolegend.com/en-us/products/alexa-fluor-488-anti-mouse-cd197-ccr7-antibody-2844
anti-Mouse CD197 (CCR7) PE (Clone 4B12) BD Biosciences 1:100 https://www.biolegend.com/en-us/products/pe-anti-mouse-cd197-ccr7-antibody-2799
anti-mouse CD279 (PD-1) PE-Cy7 (Clone 29F.1A12) Biolegend 1:400 https://www.biolegend.com/en-us/products/pe-cyanine7-anti-mouse-cd279-pd-1-antibody-7005
anti-mouse/human Bcl6 PE (Clone K112-91) BD Biosciences 1:50 https://www.bdbiosciences.com/en-us/products/reagents/flow-cytometry-reagents/research-reagents/single-color-antibodies-ruo/pe-mouse-anti-bcl-6.561522
anti-mouse/human c-Maf PE (Clone T54-853) BD Biosciences 1:200 https://www.bdbiosciences.com/en-us/products/reagents/flow-cytometry-reagents/research-reagents/single-color-antibodies-ruo/pe-mouse-anti-c-maf.565795
anti-mouse/human GL7 AF488 (Clone GL7) Biolegend 1:400 https://www.biolegend.com/en-us/products/alexa-fluor-488-anti-mouse-human-gl7-antigen-t-and-b-cell-activation-marker-antibody-9579
anti-mouse/human GL7 PerCP-Cy5.5 (Clone GL7) Biolegend 1:200 https://www.biolegend.com/en-us/products/percp-cyanine5-5-anti-mouse-human-gl7-antigen-t-and-b-cell-activation-marker-antibody-9231
anti-mouse/human GL7 PB (Clone GL7) Biolegend 1:200 https://www.biolegend.com/en-us/products/pacific-blue-anti-mouse-human-gl7-antigen-t-and-b-cell-activation-marker-antibody-9580
anti-mouse IgD AF647 (Clone 11-26c.2a) Biolegend 1:300 https://www.biolegend.com/en-us/products/alexa-fluor-647-anti-mouse-igd-antibody-4139
anti-rabbit IgG (min x-reactivity) DyLight 488 (Clone Poly4064) Biolegend 1:400 https://www.biolegend.com/en-us/products/dylight-488-donkey-anti-rabbit-igg-minimal-x-reactivity-5693
anti-mouse Ly-6C PerCP-Cy5.5 (Clone HK1.4) Biolegend 1:400 https://www.biolegend.com/en-us/products/percp-cyanine5-5-anti-mouse-ly-6c-antibody-5967
anti-mouse TCR-beta chain APC-Cy7 (Clone H57-597) BD Biosciences 1:400 https://www.bdbiosciences.com/en-us/products/reagents/flow-cytometry-reagents/research-reagents/single-color-antibodies-ruo/apc-cy-7-hamster-anti-mouse-tcr-chain.560656
anti-mouse Tigit (Vstm3) PE (Clone 1G9) Biolegend 1:200 https://www.biolegend.com/en-us/products/pe-anti-mouse-tigit-vstm3-antibody-7429
Biotin anti-mouse Tigit (Vstm3) (Clone 1G9) Biolegend 1:200 https://www.biolegend.com/en-us/products/biotin-anti-mouse-tigit-vstm3-antibody-14580
Biotin anti-mouse CD45.2 (Clone 104) Biolegend 1:400 https://www.biolegend.com/en-us/products/biotin-anti-mouse-cd45-2-antibody-5
anti-Foxo1 (Clone C29H4) Cell Signaling Technology 1:100 https://www.cellsignal.com/products/primary-antibodies/foxo1-c29h4-rabbit-mab/2880
anti-mouse Tigit (Polyclonal) R&D 1:200 https://www.rndsystems.com/products/mouse-tigit-antibody_af7267
anti-ICOS (Clone C398.4A) Biolegend 5 μg/ml https://www.biolegend.com/en-us/products/purified-anti-human-mouse-rat-cd278-icos-antibody-2477
anti-mouse/human CD3 (Clone 145-2C11) eBioscience 0.5 μg/ml https://www.thermofisher.com/antibody/product/CD3e-Antibody-clone-145-2C11-Monoclonal/16-0031-82
anti-mouse CD28 (Clone 37.51) eBioscience 1 μg/ml https://www.thermofisher.com/antibody/product/CD28-Antibody-clone-37-51-Monoclonal/16-0281-82
anti-Hamster IgG (Polyclonal) Thermo Fisher Scientific 20 μg/ml https://www.thermofisher.com/antibody/product/Goat-anti-Armenian-Hamster-IgG-H-L-Secondary-Antibody-Polyclonal/13-4113-85
Streptavidin APC Biolegend 1:500 https://www.biolegend.com/en-us/products/apc-streptavidin-1470
Streptavidin BV650 Biolegend 1:500 https://www.biolegend.com/en-us/products/brilliant-violet-650-streptavidin-7824
I-A(b) LCMV GP66-77 (DIYKGVYQKSV) tetramer-APC NIH tetramer core facility 1:100 https://tetramer.yerkes.emory.edu/reagents/3909
I-A(b) Influenza A NP311-325 (QVYSLIRPNENPAHK) tetramer-APC NIH tetramer core facility 1:100 https://tetramer.yerkes.emory.edu/reagents/3850

Eukaryotic cell lines

Policy information about cell lines and Sex and Gender in Research

Cell line source(s)	Plat-E cells were a kind of gift of Dr. Matthew Pipkin. The original source see the reference: DOI: 10.1038/sj.gt.3301206
Authentication	Plat-E cells were not authenticated.
Mycoplasma contamination	Plat-E cells were not tested for mycoplasma contamination.

Commonly misidentified lines
(See ICLAC register)

No commonly misidentified lines were used in this study.

Animals and other research organisms

Policy information about studies involving animals; ARRIVE guidelines recommended for reporting animal research, and Sex and Gender in Research

Laboratory animals	CD45.2+ C57BL/6 (Strain #: 000664), CD45.1+ C57BL/6 (Strain #: 002014), OT-II (Strain #: 004194), CD45.1+ SMARTA (Strain #: 030450), B1-8i (Strain #: 012642), and CD4-CreTg (Strain #: 017336) mice were purchased from Jackson Laboratories. CD45.2+ C57BL/6 mice were bred to CD45.1+ C57BL/6 congenic mice to generate CD45.1+CD45.2+ mice. OT-II mice were bred to CD45.1+ C57BL/6 congenic mice to generate CD45.1+CD45.2+OT-IITg and CD45.1+OT-IITg mice. Fucci2-cell cycle-reporter mice (Strain #: RBRC06511) were obtained from RIKEN and bred with OT-II mice to generate OT-IITgFucci2 mice. Bcl6f/f mice were generated at TSRI Mouse Genetics Core. Bcl6f/f mice were bred with CD4-CreTg and OT-II mice to generate OT-IITgBcl6f/fCD4-CreTg mice. Bcl6f/f mice were bred with CD4-CreTg and CD45.1+ C57BL/6 congenic mice to generate CD45.1+Bcl6f/fCD4-CreTg mice. Bcl6-protein reporter mice were generated at the Faculty of Pharmaceutical Sciences, Tokyo University of Science.
Wild animals	No wild animals were used in the study.
Reporting on sex	Experiments were conducted using age and sex matched male and female mice at 6-12 weeks of age.
Field-collected samples	No field-collected samples were used in the study.
Ethics oversight	All animals were maintained in specific pathogen–free barrier facilities and were used in accordance with protocols approved by the Institutional Animal Care and Use Committee of the University of Alabama at Birmingham (Birmingham, Alabama).

Note that full information on the approval of the study protocol must also be provided in the manuscript.

Flow Cytometry

Plots

Confirm that:

☒ The axis labels state the marker and fluorochrome used (e.g. CD4-FITC).

☒ The axis scales are clearly visible. Include numbers along axes only for bottom left plot of group (a 'group' is an analysis of identical markers).

☒ All plots are contour plots with outliers or pseudocolor plots.

☒ A numerical value for number of cells or percentage (with statistics) is provided.

Methodology

Sample preparation	Mediastinal lymph nodes and spleens were mashed through a 70 µm filter to obtain single cell suspensions. Spleen samples were further incubated with ACK lysis buffer to remove red blood cells. Cells were counted using trypan blue staining, and 3 × 106 to 5 × 106 cells were suspended in 50 µl phosphate buffered saline (PBS) containing 2% bovine serum albumin and 2 mM EDTA (FACS buffer) for staining. Nonspecific antibody binding was blocked with anti-CD16/CD32 antibodies (Biolegend) in FACS buffer for 10 minutes before staining. Dead cells were excluded through the use of a Live/Dead Fixable Dead Cell staining kit or Fixable Viability Dye eFluor 780 (Invitrogen). Allophycocyanin (APC) conjugated influenza nucleocapsid protein (NP311-325) tetramer and lymphocytic choriomeningitis virus glycoprotein (GP66-77) tetramer were provided by the NIH Tetramer Core Facility, and the staining were performed on room temperature (RT) for 1 hour. For intracellular Bcl6 and c-Maf staining, cells were fixed and permeabilized using the eBioscience Foxp3 transcription factor staining kit (Thermo Fisher Scientific). Intracellular staining was performed on ice for 45 minutes. For intracellular Foxo1 staining, cells were stimulated with 5 µg/ml anti-ICOS (Biolegend) and 0.5 µg/ml anti-CD3 (eBioscience) antibodies followed by crosslinking with 20 µg/ml goat anti-hamster IgG (MP Biomedicals) at 37°C for 30 minutes. After surface staining, cells were fixed and permeabilized using the BD Cytofix/Cytoperm™ Fixation/Permeabilization solution kit (BD Biosciences). The primary anti-Foxo1 antibody (Cell Signaling Technology) staining was performed at RT for 1 hour, and it was followed by secondary anti-rabbit IgG (minimal x-reactivity) DyLight 488 antibody (Biolegend) staining on ice for 45 minutes.
Instrument	BD LSR II, BD FACSymphony A3, BD FACSAria II, Amnis ImageStreamX Mark II
Software	Flow cytometry results were analyzed using FlowJo software (v.10.7.1). Imaging flow cytometry results were analyzed using IDEAS software (v.6.2).
Cell population abundance	Cell sorting was performed by the UAB flow cytometry core. The post-sorted population purity was tested by flow cytometry and over 95% purity. For RNA-seq, 20000 - 40000 cells were sorted directly into TRIzol-LS Reagent (Invitrogen) and RNA was isolated using miRNeasy Micro Kit (QIAGEN). For ATAC-seq, 50000 cells were sorted into 1.5 ml tubes for ATAC-seq library preparation.

Gating strategy

Based on the pattern of FSC-A/SSC-A, lymphocytes gate was used for T and B cell subsets. Single cells were gated by FSC-A/FSC-H and FSC-A/FSC-W. Live positive populations were determined by the specific antibodies, which were distinct from negative populations.

☒ Tick this box to confirm that a figure exemplifying the gating strategy is provided in the Supplementary Information.
